# Impact of Mannitol on Left Atrial Pressure in Dogs With Mitral Regurgitation

**DOI:** 10.1002/vms3.70274

**Published:** 2025-02-27

**Authors:** Jingya Yao, Takuma Aoki

**Affiliations:** ^1^ Laboratory of Small Animal Surgery Azabu University Sagamihara City Kanagawa Japan

**Keywords:** dog, left atrial pressure, mannitol, mitral regurgitation

## Abstract

**Background:**

Mannitol has been proven effective in treating cerebral oedema, owing to its osmotic diuretic effect. However, its potential to transiently increase plasma volume raises uncertainties about its impact on dogs with mitral avalve insufficiency.

**Objectives:**

This study aimed to investigate the effects of mannitol on haemodynamics and cardiac load in dogs with experimentally induced mitral regurgitation (MR), with a particular focus on the potential adverse effects of high doses of mannitol.

**Methods:**

We used implantable telemetry to monitor MR dogs for 12 consecutive hours, recording and comparing the dynamic changes in their mean left atrial pressure (mLAP) and heart rate at different doses of mannitol (low [0.5 g/kg], medium [1 g/kg] and high [2 g/kg]).

**Results:**

Compared with that in the control group, the low‐ and medium‐dose groups demonstrated a proclivity towards increased mLAP after administration, but without statistical significance. The high‐dose group exhibited an immediate increase in the mLAP after administration (*p* < 0.001), followed by a gradual return to baseline 1 h later.

**Conclusions:**

This study provides crucial reference data for the use of mannitol in treating MR. It is particularly important to exercise caution when administering high doses of mannitol to dogs with advanced stages of MR.

## Graphical abstract

In dogs with stage B mitral regurgitation (MR), low‐ and medium‐dose mannitol had minimal impact on mean left atrial pressure (mLAP) and heart rate (HR). However, high‐dose mannitol significantly increased mLAP, highlighting the importance of cautious dosing to prevent cardiac burden.

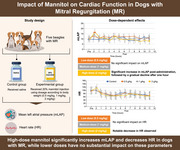

## 1 Introduction

Mitral regurgitation (MR) is a common cardiac disorder in dogs, particularly in smaller breeds and older animals, often resulting from myxomatous mitral valve disease (El Sabbagh et al. 2018). This condition is characterised by the failure of the mitral valve to close completely, causing blood to flow back into the left atrium when the left ventricle contracts (Smith et al. 2015). The backflow of blood raises the pressure in the left atrium and left ventricle, thereby increasing the cardiac workload. In some dogs, this may eventually lead to congestive heart failure (Keene et al. 2019).

**Summary**

- Mannitol effects on cardiac function in dogs with MR were evaluated.
- High‐dose mannitol significantly increased left atrial pressure in MR dogs.
- Low and medium doses of mannitol had minimal impact on heart rate and mLAP.
- Divided dosing of mannitol may reduce cardiac burden in advanced MR cases.

Mannitol is a commonly used osmotic diuretic frequently used to treat acute conditions, such as cerebral oedema, and reduce intracranial and intraocular pressure (Ballocco et al. 2019). It increases blood volume by raising plasma osmolality and promoting the movement of intracellular fluid into the blood vessels. In dogs with MR, the left atrium and left ventricle already endure significant volume overload due to the valve insufficiency. An increase in plasma volume may consequently exacerbate the cardiac burden.

Although the haemodynamic effects of mannitol have rarely been studied, a transient increase in intravascular volume has been observed in normal dogs (Cloyd et al. 1986). However, to the best of our knowledge, the impact of this transient volume increase due to mannitol use in dogs with cardiac diseases has not been thoroughly investigated. Even a temporary volume increase could have significant implications for conditions involving volume overload, such as MR. We hypothesised that in dogs with MR, administering high doses of mannitol could significantly increase cardiac burden, whereas low doses could be relatively safe and effective.

This study aimed to assess the impact of mannitol on cardiac function in dogs with MR by observing the dynamic changes in the mean left atrial pressure (mLAP) and heart rate (HR), determine the risks associated with the use of mannitol in elderly dogs with heart disease and provide insights into appropriate administration methods.

## 2 Material and Methods

### 2.1 Animals

In total, five beagles (four female dogs and one male dog), none of which had been spayed or neutered, were included in the study. Before the commencement of the experiment, several diagnostic procedures were conducted, including blood and serum biochemistry, chest radiography and echocardiography. All dogs were confirmed to be free from other conditions that might affect the experimental results. The standard of care and management of the dogs were consistent throughout all phases of the experiment. The study was approved by the Animal Experimentation Committee of Azabu University (Sagamihara City, Japan; acceptance number 230919‐13).

### 2.2 Establishment of the MR Dogs and Implantation of Transmitters

In accordance with previous reports (Ishikawa et al. 2009; Suzuki et al. 2012), MR dogs and a telemetry system for the continuous measurement of mLAP and HR were established. A sterile echocardiography probe (Vivid E9; GE Healthcare Co., Ltd., Tokyo, Japan) equipped with a 6‐MHz phased‐array transducer was used to visualise the chordae tendinae. The chordae tendinae were then cut using hooks. The mLAP was monitored in real time using a telemetry device (PhysioTel Digital M11; Data Sciences International, St. Paul, MN, USA) that had been implanted with an mLAP sensor (Andersen et al. 2017). Measurements, including HR and mLAP, were obtained and compared before and after drug administration. Postoperative chest radiography and echocardiography were performed on each dog more than 1 week after surgery and before the start of the experiment to assess the extent of MR. During surgery and within the first 12 h postoperatively, fentanyl injection (Terumo Co., Tokyo, Japan) was continuously administered at a dose of 5–10 µg/kg. For approximately 1 week after surgery, 2–4 mg/kg tramadol hydrochloride (Tramal OD, 25 mg; Nippon Shinyaku Co., Kyoto, Japan) and 15–20 mg/kg cefalexin (Larixin, 250 mg; Fujifilm Toyama Chemical Co., Tokyo, Japan) were administered twice daily. The MR model dogs were classified based on the guidelines of the American College of Veterinary Internal Medicine (Davis, CA, USA) for diagnosing and managing chronic valvular heart disease in dogs (Keene et al. 2019).

### 2.3 Dosing and Measurement

Mannitol or saline (20%) was injected into the five MR model dogs. The doses were calculated based on their body weight (0.5, 1 and 2 g/kg), with saline (5 mL/kg; dosage volume of 2 g/kg mannitol) used as a control. The drug was administered as a 15‐min continuous rate infusion, and a 7‐day washout period was allowed between each experimental dose to ensure the elimination of residual drug effects.

Each MR model dog was placed in a separate room during drug administration. To enable continuous monitoring of mLAP, a telemetry device was implanted in the left atrium of each dog. The telemetry device comprised a pressure transducer and wireless data transmission system that allowed for the real‐time recording of mLAP data while the dogs were free to move.

Using this device, we recorded the blood pressure of the experimental dogs in real time, as well as the maximum, average and minimum values of the left atrial pressure. The recorded data represent the average values from 10‐s segments in the continuous waveform recording. Measurements were obtained over a period of approximately 12.5 h, including before drug administration (the average value from 5–10 min of resting in a calm state), 15 min after the start of drug administration (i.e., post 0 h), 1 h after drug administration and every h thereafter until 12 h after drug administration.

### 2.4 Statistical Analysis

The measured values were assessed for normality using the Kolmogorov–Smirnov test and are presented as the mean ± the standard deviation. The mLAP and HR across groups receiving different doses of mannitol and the control group were compared using a linear mixed model. When significant differences in mLAP and HR were observed, they were further evaluated using the Bonferroni method for multiple comparisons. Statistical analysis was conducted using SPSS Statistics, version 22.0 (IBM Japan, Ltd., Tokyo, Japan), and a *p* value < 0.05 was considered statistically significant.

## 3 Results

The dogs weighed 10.3 ± 0.5 kg on average, with an average age of 3.2 ± 1.6 years. According to the guidelines of the American College of Veterinary Internal Medicine for diagnosing and treating chronic valvular heart disease in dogs, three dogs were classified as stage B1 (i.e., asymptomatic with no cardiac enlargement) and two dogs were classified as stage B2 (i.e., asymptomatic with cardiac enlargement; the left atrium‐to‐aorta ratio [LA/Ao] is ≥1.6 and normalised left ventricular internal diameter in end‐diastole [LVIDDN] is ≥1.7). The average LA/Ao and LVIDDN of all five dogs were 1.62 ± 0.10 and 1.65 ± 0.3, respectively. No clinical abnormalities were observed in any of the dogs after the administration of mannitol.

Tables 1 and 2 show the mean values ± 95% confidence intervals and *p‐*values for mLAP (Table 1) and HR (Table 2) in the mannitol‐treated groups at each dose and the control group treated with saline.

**TABLE 1:** Mean left atrial pressure (mLAP): comparison with the control group, using linear mixed models.

	Control	Low‐dose mannitol	Appropriate‐dose mannitol	High‐dose mannitol	Control	Low‐dose mannitol	Appropriate‐dose mannitol
mLAP	Mean ± 95% CI	Mean ± 95% CI	*p* value	Mean ± 95% CI	*p* value	Mean ± 95% CI	*p* value
Pre	9.4 ± 2.73	10.6 ± 2.73	> 0.999	10.6 ± 2.73	> 0.999	9.4 ± 2.73	> 0.999
Post 0 h	9.6 ± 2.73	12.6 ± 2.73	0.32	13.8 ± 2.73	0.073	16.6 ± 2.73	**0.001**
Post 1 h	9.4 ± 2.73	10.6 ± 2.73	> 0.999	10.2 ± 2.73	> 0.999	11.2 ± 2.73	0.996
Post 2 h	9.8 ± 2.73	12.4 ± 2.73	0.485	11 ± 2.73	> 0.999	10.6 ± 2.73	> 0.999
Post 3 h	9.6 ± 2.73	11.2 ± 2.73	> 0.999	9.6 ± 2.73	> 0.999	8.6 ± 2.73	> 0.999
Post 4 h	12.8 ± 2.73	12.8 ± 2.73	> 0.999	9.2 ± 2.73	0.16	12.6 ± 2.73	> 0.999
Post 5 h	11 ± 2.73	12.4 ± 2.73	> 0.999	11.2 ± 2.73	> 0.999	9.6 ± 2.73	> 0.999
Post 6 h	11.8 ± 2.73	12.2 ± 2.73	> 0.999	9.4 ± 2.73	0.589	9.2 ± 2.73	0.485
Post 7 h	8.4 ± 2.73	12.2 ± 2.73	0.124	10.2 ± 2.73	0.996	9.4 ± 2.73	> 0.999
Post 8 h	8.6 ± 2.73	12.6 ± 2.73	0.096	10.6 ± 2.73	0.844	9 ± 2.73	> 0.999
Post 9 h	9 ± 2.73	10.4 ± 2.73	> 0.999	10.8 ± 2.73	0.996	7.6 ± 2.73	> 0.999
Post 10 h	11.8 ± 2.73	10 ± 2.73	0.996	9.8 ± 2.73	0.844	8.6 ± 2.73	0.256
Post 11 h	10.8 ± 2.73	11 ± 2.73	> 0.999	12.2 ± 2.73	> 0.999	7.6 ± 2.73	0.256
Post 12 h	8.6 ± 2.73	8.6 ± 2.73	> 0.999	10 ± 2.73	> 0.999	8.6 ± 2.73	> 0.999

*Note*: The mean and 95% confidence interval (95% CI) of mLAP and Bonferroni‐adjusted significance levels are shown. Significant values are indicated in bold font.

**TABLE 2:** Heart rate (HR): comparison with the control group, using linear mixed models.

	Control	Low‐dose mannitol		Appropriate‐dose mannitol		High‐dose mannitol	
HR	Mean ± 95% CI	Mean ± 95% CI	*p* value	Mean ± 95% CI	*p* value	Mean ± 95% CI	*p* value
Pre	101.2 ± 21.79	95.4 ± 21.79	> 0.999	100.2 ± 21.79	> 0.999	101.2 ± 21.79	> 0.999
Post 0 h	101 ± 21.79	102.8 ± 21.79	> 0.999	101.2 ± 21.79	> 0.999	116.2 ± 21.79	0.116
Post 1 h	89.6 ± 21.79	86 ± 21.79	> 0.999	78.2 ± 21.79	0.36	76.8 ± 21.79	0.243
Post 2 h	79 ± 21.79	80.4 ± 21.79	> 0.999	67.8 ± 21.79	0.379	68.2 ± 21.79	0.422
Post 3 h	68.8 ± 21.79	72 ± 21.79	> 0.999	64 ± 21.79	> 0.999	65.8 ± 21.79	> 0.999
Post 4 h	69.8 ± 21.79	68.8 ± 21.79	> 0.999	65.2 ± 21.79	> 0.999	66 ± 21.79	> 0.999
Post 5 h	83.6 ± 21.79	75 ± 21.79	0.72	75.2 ± 21.79	0.754	67.8 ± 21.79	0.095
Post 6 h	88.2 ± 21.79	85.6 ± 21.79	> 0.999	82.2 ± 21.79	> 0.999	70 ± 21.79	**0.04**
Post 7 h	76.8 ± 21.79	77.6 ± 21.79	> 0.999	83.4 ± 21.79	> 0.999	79.4 ± 21.79	> 0.999
Post 8 h	70 ± 21.79	76.8 ± 21.79	> 0.999	83.4 ± 21.79	0.203	73.6 ± 21.79	> 0.999
Post 9 h	74.8 ± 21.79	71 ± 21.79	> 0.999	74 ± 21.79	> 0.999	70 ± 21.79	> 0.999
Post 10 h	74.8 ± 21.79	68.2 ± 21.79	> 0.999	65.4 ± 21.79	0.598	64.4 ± 21.79	0.467
Post 11 h	68.2 ± 21.79	68.8 ± 21.79	> 0.999	68.2 ± 21.79	> 0.999	65.6 ± 21.79	> 0.999
Post 12 h	70 ± 21.79	71.4 ± 21.79	> 0.999	63 ± 21.79	> 0.999	69.6 ± 21.79	> 0.999

*Note*: The mean and 95% confidence interval (95% CI) of the HR and Bonferroni‐adjusted significance levels are shown. Significant values are indicated in bold font.

**FIGURE 1:**
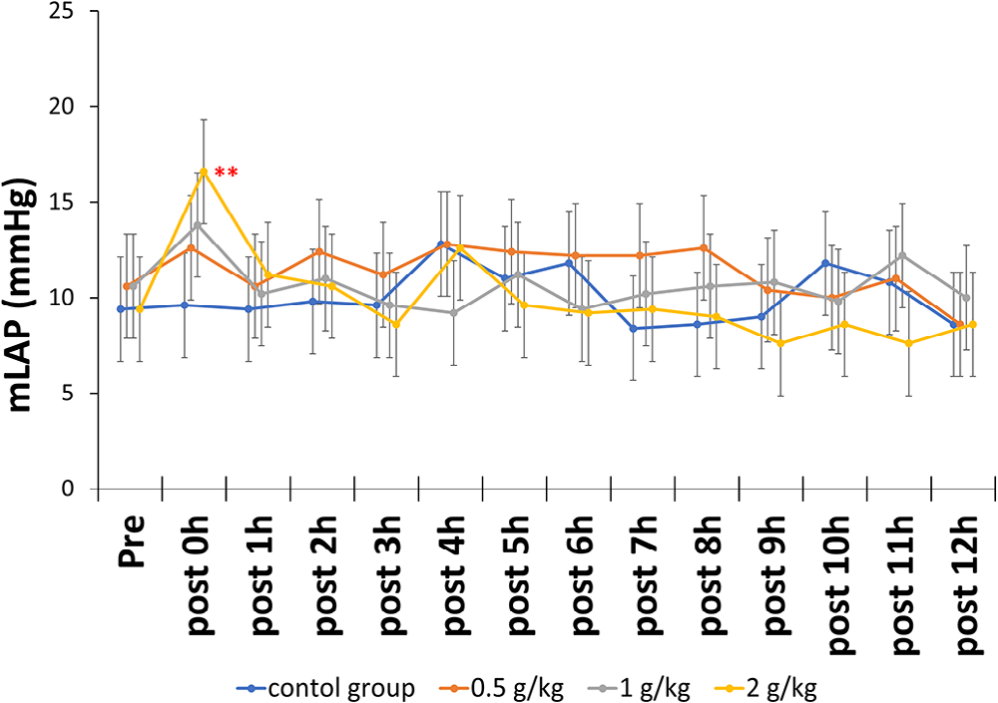
Mean left atrial pressure (mLAP) after 15 min of continuous rate infusion of mannitol in five dogs. The graph represents the mean values, with bars indicating the 95% confidence intervals. **p < 0.01 versus control.

Figure 1 depicts alterations in the mLAP. The mLAP in the low‐dose group (0.5 g/kg) and medium‐dose group (1 g/kg) exhibited a slight upward trend 15 min after the start of drug administration (i.e., post 0 h), although these changes were not statistically significant. However, the high‐dose group (2 g/kg) demonstrated a significant increase in the mLAP 15 min after drug administration (*p* < 0.001), followed by a gradual return to baseline 1 h later.

**FIGURE 2:**
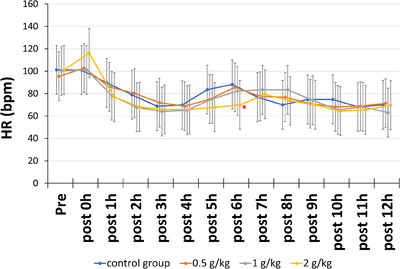
Changes in the heart rate of the five dogs from before to 12 h after drug administration. The graph presents the mean values, with bars representing the 95% confidence intervals. *p < 0.05 versus control.

Figure 2 illustrates the changes in HR among five MR model dogs from pre‐dosing to 12 h post‐administration. HRs in the low‐dose (0.5 g/kg) and medium‐dose (1 g/kg) groups did not significantly differ from those in the control group. However, in the high‐dose group (2 g/kg), although there was a tendency for HR to increase 15 min after drug administration (*p* = 0.116), a significant decrease in HR was observed at the 6th h, which was lower than that in the control group (*p* = 0.04).

## 4 Discussion

The findings of this study indicated that mannitol at varying doses exerts different effects on mLAP and HR in dogs with experimental MR. Specifically, the low (0.5 g/kg) and medium (1 g/kg) doses of mannitol both demonstrated no significant impact on mLAP and HR. This finding indicated that, at these doses, mannitol has a minimal impact on cardiac burden and a small effect on cardiac function in MR dogs. However, the high dose (2 g/kg) of mannitol significantly increased mLAP 15 min after the start of drug administration in the MR dogs. However, mLAP returned to baseline 1 h later. These results indicated that high doses of mannitol substantially increase the cardiac burden in a relatively short and transient period.

In a previous study on normal dogs, mannitol at doses of 0.5 g/kg and 1.0 g/kg did not affect the central compartment volume, but a significant increase was observed at 1.5 g/kg (Cloyd et al. 1986). This finding indicated that administering high doses of mannitol leads to a swift, uncompensated movement of water from the intracellular to the extracellular space. Furthermore, even with the administration of mannitol at a relatively low dose of 0.8 g/kg, plasma volume in healthy dogs increased by 20% 15 min post‐administration (Robinson et al. 2011). Although plasma volume tends to decrease with increased urine output, it remained elevated for up to 120 min following administration. Therefore, in MR dogs, as observed in the current study, high doses of mannitol may pose a significant cardiac burden.

In dogs, pulmonary oedema may occur when the mean pulmonary capillary wedge pressure exceeds 24 mmHg (Guyton and Lindsey 1959). As diagnostic imaging was not performed because of the good clinical condition of the dogs in this study, whether true pulmonary oedema occurred remains uncertain. However, despite the use of high‐dose mannitol, the left atrial pressure was 16.6 ± 2.7 mmHg, suggesting that it may not have reached levels sufficient to induce pulmonary oedema. Nevertheless, as the dogs were classified as stage B, caution should be exercised when clinically using high‐dose mannitol in dogs with more advanced MR. If high‐dose mannitol administration is deemed necessary, consideration should be given to divided dosing to minimise acute effects on left atrial pressure and avoid increasing cardiac workload. In this study, despite high‐dose mannitol administration, left atrial pressure returned to pre‐administration levels 1 h later. Therefore, dividing the dose, such as administering half the dose every hour, may enhance safety when using high‐dose mannitol. As this study involved single‐dose administration, future research should investigate the impact on left atrial pressure when mannitol is administered in divided doses over multiple administrations.

The low‐dose and medium‐dose groups showed no change in HR, whereas the high‐dose group showed increased HR 15 min after drug administration. With a 3 g/dL dose of mannitol, the heart rate of healthy dogs increased within 30 min and then decreased by 60 min post‐administration (Kambayashi et al. 2023). In our study, a higher dose of mannitol was also associated with an elevated heart rate, showing an average increase of approximately 15 bpm 15 min post‐administration. The osmotic increase caused by mannitol is thought to induce the release of nitric oxide from vascular endothelial cells, leading to vasodilation and stimulation of the sympathetic nervous system(Kambayashi et al. 2023. Therefore, high‐dose mannitol administration may temporarily increase the heart rate in dogs. However, HR significantly decreased 6 h after drug administration. In general, in conditions such as haemorrhage, in which the intravascular fluid volume decreases and blood pressure drops, sympathetic nervous system activation typically increases the HR (Woodman et al. 1986). When an intravenous infusion of 31.4 to 44.0 mL/kg of saline was administered over 3 to 15 min to normal dogs, the heart rate increased from an average of 73 to 148 bpm (Vatner et al. 1975). Although this dose is more than six times the amount used in our study, the increased right atrial pressure due to the high dose of saline activated the Bainbridge reflex, leading to an increase in heart rate. This increase in heart rate persisted for 150 min without any decrease owing to the baroreceptor reflex.

Following the administration of 1 g/kg (5 mL/kg) 20% mannitol to dogs, the blood sodium level decreased at 5 min but normalised by 60 min (Hoehne et al. 2021), suggesting that the significant increase in intravascular volume is transient. Co‐administration of high‐dose saline and pressor agents resulted in a decrease in heart rate owing to the baroreceptor reflex (Vatner et al. 1975). In this study also, it is possible that the transient preload caused by volume loading (10 mL/kg 20% mannitol), coupled with an increase in blood pressure, led to the activation of the baroreceptor reflex. However, as peripheral vascular resistance was not measured in this study, the cause of the decrease in heart rate could not be determined.

This study has limitations. First, it utilised a young dog model of MR, and whether older dogs with naturally occurring MR would tolerate high‐dose mannitol similarly is unclear. Second, the distribution of stages in dogs with naturally occurring MR shows that 65% are in stage B1 and 15% are in stage B2, suggesting some extrapolation of the results. However, stage C, which indicates pulmonary oedema, is present in 20% of cases (Franchini et al. 2021). This study did not examine the impact of high‐dose mannitol in dogs with a history of pulmonary oedema. Third, this study was based on the single‐dose administration of mannitol; thus, the effects of repeated dosing in MR dogs were not explored. The mLAP tended to be higher in the low‐dose and medium‐dose groups than in the control group. However, a significant decrease in HR was observed only in the high‐dose group. Therefore, that the decrease in HR may have been a type I error due to multiple comparisons cannot be ruled out, even after applying the Bonferroni correction. Finally, although the high‐dose group showed a significant increase in left atrial pressure, it did not reach levels that would induce pulmonary oedema, and the clinical condition of the subjects remained stable. Imaging evaluations consequently were not conducted. To objectively assess the presence of pulmonary oedema, chest radiography should have been performed.

## 5 Conclusions

This study provides crucial reference data for the utilisation of mannitol in treating MR. It is important to exercise caution when administering high doses of mannitol to dogs with advanced stages of MR. Future studies should investigate the effects of varying doses and modes of administration on the long‐term prognosis of dogs with spontaneous MR.

## Author Contributions

**Jingya Yao**: data curation, formal analysis, investigation, methodology, project administration, visualisation, writing – original draft. **Takuma Aoki**: conceptualization, formal analysis, investigation, methodology, project administration, supervision, validation, visualisation, writing – review and editing.

## Ethics Statement

The authors affirm adherence to the journal's ethical policies, as outlined on the author guidelines page of the journal. The study was approved and conducted in accordance with guidelines for the Animal Experiment Committee of the Azabu University (Sagamihara City, Japan; Approval No. 230919‐13).

## Conflicts of Interest

The authors declare no conflicts of interest.

### Peer Review

The peer review history for this article is available at https://www.webofscience.com/api/gateway/wos/peer‐review/10.1002/vms3.70274.

## Data Availability

The data underpinning the findings of this study can be obtained from the corresponding author upon reasonable request.

## References

[vms370274-bib-0001] Andersen, N. K. , O. Meyer , A. Bradley , et al. 2017. “Evaluation of the PhysioTel™ Digital M11 Cardiovascular Telemetry Implant in Socially Housed Cynomolgus Monkeys up to 16 Weeks After Surgery.” Journal of Pharmacological and Toxicological Methods 87: 82–92. 28416413 10.1016/j.vascn.2017.04.007

[vms370274-bib-0002] Ballocco, I. , M. A. Evangelisti , R. Deiana , et al. 2019. “A Pilot Study Evaluating the Effect of Mannitol and Hypertonic Saline Solution in the Treatment of Increased Intracranial Pressure in 2 Cats and 1 Dog Naturally Affected by Traumatic Brain Injury.” Journal of Veterinary Emergency and Critical Care 29: 578–584. 31448527 10.1111/vec.12880

[vms370274-bib-0003] Cloyd, J. C. , B. D. Snyder , B. Cleeremans , S. R. Bundlie , C. H. Blomquist , and D. J. Lakatua . 1986. “Mannitol Pharmacokinetics and Serum Osmolality in Dogs and Humans.” Journal of Pharmacology and Experimental Therapeutics 236: 301–306. 3080582

[vms370274-bib-0004] El Sabbagh, A. , Y. Reddy , and R. Nishimura . 2018. “Mitral Valve Regurgitation in the Contemporary Era: Insights Into Diagnosis, Management, and Future Directions.” Journal of the American College of Cardiology: Cardiovascular Imaging 11: 628–643. 29622181 10.1016/j.jcmg.2018.01.009

[vms370274-bib-0005] Franchini, A. , M. Borgarelli , J. A. Abbott , et al. 2021. “The Longitudinal Outcome of Canine (K9) Myxomatous Mitral Valve Disease (LOOK‐Mitral registry): Baseline Characteristics.” Journal of Veterinary Cardiology 36: 32–47. 34062479 10.1016/j.jvc.2021.04.005

[vms370274-bib-0006] Guyton, A. C. , and A. W. Lindsey . 1959. “Effect of Elevated Left Atrial Pressure and Decreased Plasma Protein Concentration on the Development of Pulmonary Edema.” Circulation Research 7: 649–657. 13663218 10.1161/01.res.7.4.649

[vms370274-bib-0007] Hoehne, S. N. , I. D. Yozova , B. Vidondo , and K. N. Adamik . 2021. “Comparison of the Effects of 7.2% Hypertonic Saline and 20% Mannitol on Electrolyte and Acid‐Base Variables in Dogs With Suspected Intracranial Hypertension.” Journal of Veterinary Internal Medicine 35: 341–351. 33236379 10.1111/jvim.15973 PMC7848367

[vms370274-bib-0008] Ishikawa, T. , R. Tanaka , S. Suzuki , et al. 2009. “Daily Rhythms of Left Atrial Pressure in Beagle Dogs With Mitral Valve Regurgitation.” Journal of Veterinary Internal Medicine 23: 824–831. 19496915 10.1111/j.1939-1676.2009.0322.x

[vms370274-bib-0009] Kambayashi, R. , H. Izumi‐Nakaseko , A. Goto , et al. 2023. “Both Osmolality‐Dependent and Independent Mechanisms Are Associated With Acute Hyperglycemia‐Induced Cardiovascular Adverse Reactions: Analysis of the Mutual Interactions Leading to Cardiovascular Phenotypes in Dogs.” Journal of Toxicological Sciences 48: 169–178. 36858642 10.2131/jts.48.169

[vms370274-bib-0010] Keene, B. W. , C. E. Atkins , J. D. Bonagura , et al. 2019. “ACVIM Consensus Guidelines for the Diagnosis and Treatment of Myxomatous Mitral Valve Disease in Dogs.” Journal of Veterinary Internal Medicine 33: 1127–1140. 30974015 10.1111/jvim.15488 PMC6524084

[vms370274-bib-0011] Robinson, R. , I. Schwendenwein , S. Wacek , B. Nell , and M. Mosing . 2011. “Plasma Volume and Electrolyte Changes Following Intravenous Infusion of Hypertonic Hydroxyethyl Starch Versus Mannitol in Healthy Dogs.” Veterinary Journal 190: 268–272. 21112802 10.1016/j.tvjl.2010.10.019

[vms370274-bib-0012] Smith, F. W. , L. P. Tilley , M. Oyama , and M. M. Sleeper . 2015. Manual of Canine and Feline Cardiology. 4th ed. Elsevier Health Sciences.

[vms370274-bib-0013] Suzuki, S. , R. Fukushima , T. Ishikawa , et al. 2012. “Comparative Effects of Amlodipine and Benazepril on Left Atrial Pressure in Dogs With Experimentally‐Induced Mitral Valve Regurgitation.” BMC Veterinary Research 8: 166. 22989022 10.1186/1746-6148-8-166 PMC3489586

[vms370274-bib-0014] Suzuki, S. , T. Ishikawa , L. Hamabe , et al. 2011. “The Effect of Furosemide on Left Atrial Pressure in Dogs With Mitral Valve Regurgitation.” Journal of Veterinary Internal Medicine 25: 244–250. 21314718 10.1111/j.1939-1676.2010.0672.x

[vms370274-bib-0015] Vatner, S. F. , D. H. Boettcher , G. R. Heyndrickx , and R. J. McRitchie . 1975. “Reduced Baroreflex Sensitivity With Volume Loading in Conscious Dogs.” Circulation Research 37: 236–242. 1149198 10.1161/01.res.37.2.236

[vms370274-bib-0016] Woodman, O. L. , J. Amano , T. H. Hintze , and S. F. Vatner . 1986. “Augmented Catecholamine Uptake by the Heart During Hemorrhage in the Conscious Dog.” American Journal of Physiology 250: H76–H81. 3942240 10.1152/ajpheart.1986.250.1.H76

